# Zero-shot model-free learning of periodic movements for a bio-inspired soft-robotic arm

**DOI:** 10.3389/frobt.2023.1256763

**Published:** 2023-10-19

**Authors:** Paris Oikonomou, Athanasios Dometios, Mehdi Khamassi, Costas S. Tzafestas

**Affiliations:** ^1^ Division of Signals, Control and Robotics, School of Electrical and Computer Engineering, National Technical University of Athens, Athens, Greece; ^2^ Sorbonne Université, Centre National de la Recherche Scientifique, Institute of Intelligent Systems and Robotics, Paris, France

**Keywords:** robot learning, motion control, probabilistic movement primitives, central pattern generators, soft robots

## Abstract

In recent years, soft robots gain increasing attention as a result of their compliance when operating in unstructured environments, and their flexibility that ensures safety when interacting with humans. However, challenges lie on the difficulty to develop control algorithms due to various limitations induced by their soft structure. In this paper, we introduce a novel technique that aims to perform motion control of a modular bio-inspired soft-robotic arm, with the main focus lying on facilitating the qualitative reproduction of well-specified periodic trajectories. The introduced method combines the notion behind two previously developed methodologies both based on the Movement Primitive (MP) theory, by exploiting their capabilities while coping with their main drawbacks. Concretely, the requested actuation is initially computed using a Probabilistic MP (ProMP)-based method that considers the trajectory as a combination of simple movements previously learned and stored as a MP library. Subsequently, the key components of the resulting actuation are extracted and filtered in the frequency domain. These are eventually used as input to a Central Pattern Generator (CPG)-based model that takes over the generation of rhythmic patterns at the motor level. The proposed methodology is evaluated on a two-module soft arm. Results show that the first algorithmic component (ProMP) provides an immediate estimation of the requested actuation by avoiding time-consuming training, while the latter (CPG) further simplifies the execution by allowing its control through a low-dimensional parameterization. Altogether, these results open new avenues for the rapid acquisition of periodic movements in soft robots, and their compression into CPG parameters for long-term storage and execution.

## 1 Introduction

Bioinspired robotics research constantly explores solutions inspired by biology to improve the materials, the behavior and the adaptivity of robots, trying to approach the combined efficiency, flexibility and low computational cost of biological organisms (Meyer et al., 2005; Pfeifer et al., 2007; Meyer and Guillot, 2008; Floreano et al., 2014; Ijspeert, 2014; Renaudo et al., 2014). Among the explored solutions, soft robots constitute a promising field of research to come up with compliant and flexible robots in unpredictable environments, as well as for applications requiring safe physical interactions with humans (Kim et al., 2013). Nevertheless, it remains challenging to endow these robots with efficient control algorithms for real-world applications, due to the difficulty to model their behaviour. In particular, we are interested here in healthcare applications where replicating human gestures with a soft robot could enable safe assistance at home to the disabled or non-autonomous elderly people.

Well organized healthcare systems are increasing the life expectancy of modern societies, according to World Health Organization’s research on health and aging (World Health Organization, 2015). There is a significant percentage of population with special requirements for nursing attention. Healthcare experts are supporting these people during the performance of Activities of Daily Living (ADL) such as showering and eating, inducing increased workload and demand for skilled personnel. Personal care (showering or bathing) is included among the first ADL, which incommodate an elderly’s life (Millán-Calenti et al., 2010), representing the strongest predictor of subsequent institutionalization (Fong et al., 2015). The robotics community continuously undertake research to develop solutions supporting everyday tasks in both in-house and clinical environments (Driessen et al., 2001; Hirose et al., 2012).

Specifically in the context of an assistive bathing robot, which requires the execution of proper interactive tasks, the expertise of clinical personnel should also be integrated into the behaviour of the robotic system, in order to execute proper washing actions in a human-friendly way, increasing the comfort of an elderly user. Hence, learning of bathing motions from expert’s demonstration is required, a process that might raise specific requirements for the robot, in terms of execution time and motion complexity.

### 1.1 Introduction to soft robots

**FIGURE 1:**
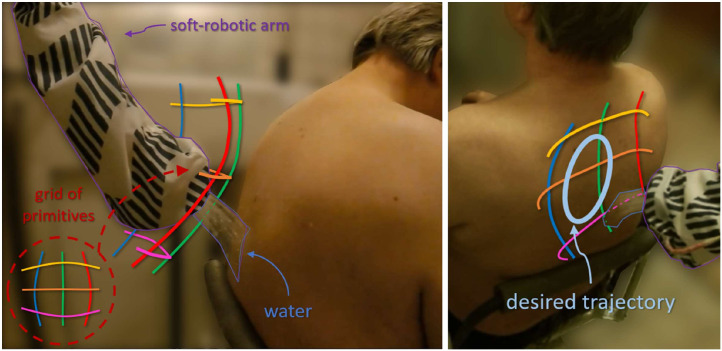
The soft robotic arm covered with nylon ensuring water resistance, while performing water pouring (part of the showering task) in a clinical environment. The colored lines depict the mean value of the learned ProMPs in the task space, covering the desired subspace of the robot’s workspace. A projection of the grid of primitives on the human back is also illustrated at the bottom left of the figure. Adapted from Oikonomou et al. (2022), with permission from IEEE.

Such an interactive bathing application, which might involve robotic interaction with the human, is way more demanding in terms of safety than other assistive robotics tasks. Over the last years, research effort has been focused on continuum bio-inspired manipulators based on soft robotic technologies (Laschi et al., 2016). The inherent or structural compliance of these technologies gives them the ability to actively interact with the environment with drastically reduced risks of injuries (e.g., in medical applications), and provides flexibility in environments where a target is unreachable by rigid arms. Many continuum manipulators have already been presented with tendon (Renda and Laschi, 2012) or pneumatic actuation (Grzesiak et al., 2011) or a combination of those (Ansari et al., 2017). Part of this research effort contributed to the recent developments of the I-SUPPORT project (EU H2020 Grant Agreement no. 643666) (Zlatintsi et al., 2020) (Figure 1), aiming at conceiving an innovative, modular robotic system for the support of frail older adults to safely and independently complete various physically and cognitively demanding bathing tasks, such as properly washing their back and their lower limbs.

Despite their natural compliance and biomorphic control properties, all these systems present specific problems and limitations that still prevent their widespread use in many application domains. These challenges are mostly related to the difficulty regarding the computation of a mathematical model that simulates the system’s dynamics. Hence, the design of efficient control schemes based on classical methods that can provide accuracy and robustness in dynamic tasks, is not straightforward. As a consequence, more sophisticated kinematic analysis is required, due to the non-linearity of the mechanical and actuation structure, as well as the high complexity introduced by the multiple degrees of freedom.

### 1.2 Control strategies developed for soft robots

To address these issues, analytic kinematic models based on constant curvature assumption have been established (Webster and Jones, 2010), and powerful control strategies for continuum manipulators are still being developed (George Thuruthel et al., 2018). Recent model-based approaches like the one introduced in Kapadia et al. (2010) are specifically developed to perform dynamic motion control on continuum robots with certain properties, such as extension, contraction, or omnidirectional bending. In Falkenhahn et al. (2017), suitable models were developed with a combination of feed-forward control and decoupled PD-controllers, and applied to a pneumatically actuated manipulator. A different approach based on open-loop predictive controllers was proposed in George Thuruthel et al. (2017), using machine learning-derived dynamic models directly from the actuation to the task space. The work presented in Godage et al. (2016) was based on a different set of techniques, in which novel spatial dynamics were applied to variable length multi-section continuum arms under the assumption of circular arc deformation of continuum sections without torsion. A relevant approach is presented in Braganza et al. (2007) where the authors used a feed-forward neural network component to compensate for dynamic uncertainties.

### 1.3 Related work

From the literature review in the previous section, we could see that the control schemes proposed for soft robots are highly dependent on the hardware set-up and actuation. Thus, attempting a fair performance comparison, we focus on dynamic control strategies applied onto the same soft robot. The algorithmic elements vary from Reinforcement Learning (RL) to motor control-based methods (e.g., Central Pattern Generators (CPGs) and Probabilistic Movement Primitives (ProMPs)) or a combination of them. But before that, some of the challenges of transferring the ProMP framework from rigid to soft robots are presented.

*ProMPs on rigid robots:* In some works where ProMPs have been applied to rigid robots (Paraschos et al., 2013; Paraschos et al., 2018; Gomez-Gonzalez et al., 2020), the generation of primitive demonstrations has been done through kinesthetic teaching of the robot by a user. At the same time, the static mapping between the task and the joint space is given by a mathematical model based on the known geometry of the rigid manipulator, e.g., the analytical solution of the inverse kinematics. However, in the present application, kinesthetic teaching is not feasible, and the mapping between task and joint space is not provided due to the complex mechanical structure of the soft robot. Hence, the generation of demonstrations as well as the computation of inverse kinematics should be redefined.

*Interactive Dynamic Movement Primitives for replicating human demonstrations:* A methodology that partially shares the same objectives with the present work, but be developed and tested on a rigid robot, was introduced in Dometios et al. (2018). There, the authors proposed an interactive version of Dynamic Movement Primitives accompanied by a vision-based controller with the aim of reproducing demonstrated washing actions while also adapting the motion of the robot’s end-effector on the moving user’s body parts. However, this work focuses only on planning of periodic motions in the task-space and does not take into account the motion control aspect of the soft-robotic arm.

*CPG-based approach for periodic movements:* A relevant work introduced in Oikonomou et al. (2020) presented a model-free neurodynamic controller based on CPGs. The goal was the generation and tracking of rhythmic motion patterns of desired features, executed by the end-effector (EE) of a single-module version of the soft-robotic arm described in Section 2. In the present work, a zero-shot learning approach is implemented that provides access for execution to almost any rhythmic motion, and avoids the time-consuming training process required in Oikonomou et al. (2020). In addition, we focus on a more robust implementation that expands the workspace of the soft arm and the variability of the feasible periodic movements. This is achieved by adding an extra module on the robot which results in the increase of the actuation space. An extended comparison between the two approaches in the generation of rhythmic motions is given in Section 5.4.

*Model-Based RL for Closed-Loop Dynamic Control:* In Thuruthel et al. (2019) a closed-loop predictive controller was implemented and evaluated on similar hardware. There, a model-based policy learning algorithm was trained through trajectory optimization and supervised learning. The focus of this approach was to achieve trajectory tracking accuracy at each time step, a requirement rarely set for soft robots. Such control schemes require large amount of data, high computational resources and many iterations for successfully reproducing a single trajectory, implying time-consuming training phase until convergence is reached. Moreover, the temporal scalability of this approach, meaning the capability to execute trajectories with specific timing properties, is not evident.

*ProMP-based approach for movements of single direction:* In another work presented in Oikonomou et al. (2021), the proposed controller used ProMPs to create a mapping at the primitive level between task- and actuation-space. The proper combination of ProMPs aimed for the reproduction of a trajectory defined by sparse way-points with time-constraints. At the same time, the unknown inverse kinematics of the robot are approximated by an RL-based algorithm. Despite its online adaptability, this approach does not exploit sufficiently the available information, but focuses only on the points of high interest - the so-called conditioning points - which are sparse. Hence, the trained model is rarely updated. Another flaw of the methodology is that the requested trajectories are limited to be similar with the derived primitive demonstrations, e.g., in terms of trajectory direction/flow.

*ProMP-based approach for arbitrary movements:* To cope with the drawback of the last approach, in Oikonomou et al. (2022) the authors proposed a method for the qualitative reproduction of human demonstrations. This work considers that a complex trajectory could be derived from the proper composition and asynchronous sequential and/or parallel activation of learned parameterizable simple movements that constitute a knowledge base. This approach exploited the mapping at the primitive-level provided by the ProMP framework to transfer the composition’s parameters unchanged into the actuation space for execution. This simplification of the trajectory control task proved to be useful for robots of complex unmodeled dynamics. Even though the presented method already constituted a zero-shot approach for the qualitative reproduction of any trajectory, it lacks the capability to efficiently modulate the features of complex rhythmic motions; these can be tuned out of simple commands in the actuation space, as seen in Oikonomou et al. (2020). Moreover, in Oikonomou et al. (2022) the complexity of the output - that is, a linear asynchronous combination of multiple ProMPs - results in increasing the required computational effort, as well as in abrupt changes of velocity. Eventually, the online transition to different rhythmic patterns is not smooth. A more detailed comparison between Oikonomou et al. (2022) and the proposed methodology is given in Section 5.3, where the necessity of the present work to handle periodic trajectories is highlighted through novel experiments.

### 1.4 Contribution

A soft robot like the one examined in the present work (see Section 2) is not often assigned with the task of path following with high-precision; the mechanical properties of its design are mostly exploited in tasks where safety must be ensured through compliance, such as those involving human-robot interaction or manipulation of fragile materials. The task examined in this paper does not differ from this class of applications. Hence our focus lies on the qualitative reproduction of trajectories, rather than on high-precision replication.

In this paper, we present an architecture that aims to perform motion control on a two-module bio-inspired soft-robotic arm. Particularly, it focuses on the qualitative reproduction of rhythmic patterns that are characterized by specific features defined by a user. The designed controller is built upon the methodology introduced in Oikonomou et al. (2022) where the requested trajectory is decomposed into simpler movements by exploiting the enhanced parameterizability of ProMPs. The extension proposed in the present work estimates the key features of the required oscillation at the actuation level, through a Fourier transformation applied onto the signals derived from the previous step. Then, a CPG model is assigned with the task to generate oscillatory motion of appropriate features on the motors. The efficiency of the proposed methodology is evaluated on a real soft-robotic arm. The experimental results highlight its advantages over other methods (Section 1.3) when reproducing rhythmic motion patterns using a complex robotic system with unknown dynamics, in applications where high-precision is not required.

The key novelties of this paper are summarized below.

• reproduction of rhythmic patterns of desired features with a soft robot;
• a generic model-free method that can be applied to any robot;
• fast training on the real robot, avoiding any simulation;
• zero-shot learning - execution of unseen trajectories right at first execution;
• easy modulation of periodic motion’s features (amplitude, *etc.*) through a CPG model.

### 1.5 Paper structure

Although the proposed methodology is model-free - not model-based - and could be applied to any robot, thedescription of the specific robotic device (Section 2) is necessary to take place before the methodology, so that the latter (Sections 3 and 4) is comprehensive to the reader. A detailed presentation of the ProMP-based control architecture for non-periodic trajectories is given in Section 3, and the corresponding extension for periodic motions using CPGs in Section 4. Experimental evaluation results are presented in Section 5, while concluding remarks along with indicative future research directions are provided in Section 6.

## 2 The soft-robotic arm

Bio-inspired robots are those that draw inspiration from some biological systems (animals, plants, *etc.*) in many aspects, such as in their design, behavior and operation (Floreano et al., 2014). Lately, these robots have gained increasing attention due to their capability to be adaptable, compliant and energetically efficient. The soft-robotic arm used in our work follows bio-inspired design principles building upon prior soft-bodied robot design concepts (Manti et al., 2017), such as the octopus-like robot presented in Laschi et al. (2012); particularly, its soft structure that provides the advantage of natural compliance, as well as its multi-modularity that allows the application of bio-inspired control methods, such as the CPGs studied on the salamander (Chevallier et al., 2008). Such modular designs take also inspiration from biological structures (Trivedi et al., 2008).

**FIGURE 2:**
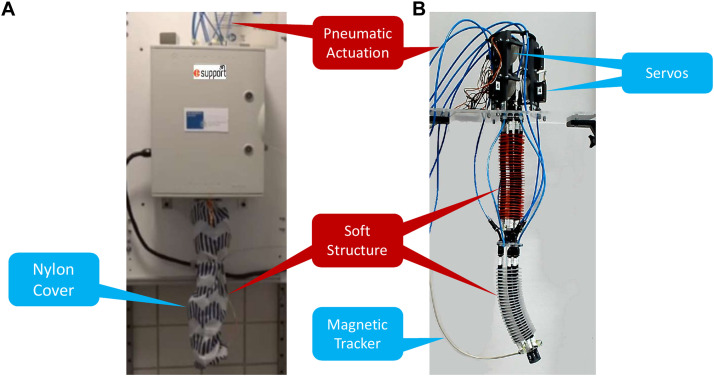
The two module ‘soft-robotic arm developed during the i-Support project, **(A)** installed in a clinical environment and **(B)** a replicated version in the lab. Adapted from Oikonomou et al. (2021) with permission from IEEE.

Specifically, the design and structure of the two-module soft manipulator (Figure 2) is analytically described in Ansari et al. (2017) and Manti et al. (2016). In a nutshell, our robotic module has been adapted from the soft arm which was developed by the Biorobotics Institute (Pisa, Italy) in the frame of the EU project I-SUPPORT. Each module comprising the robot is made up of hybrid actuation. At first, three radially symmetric tendons driven by three servomotors (Hitec HS-422 Super Sport - Supermodified Servo by 01™ Mechatronics) change the configuration of the module after modifying their cable’s length resulting in extension, contraction, or omnidirectional bending. These are combined with pneumatic chambers whose actuation is considered to be fixed in this work. Therefore, the real robot actuation is based on six inputs at the motor control level. Regarding position feedback, a 3D magnetic tracker (3D Guidance trakSTAR Class 1 Type B by Ascension Technology Corp.) was used, whose 6-DoF electromagnetic probe is attached at the end of the manipulator, providing its position and orientation.

Similar to other bio-inspired systems, the soft-robotic arm of Figure 2 suffers from various limitations induced by its soft structure’s properties, such as the infinite degrees of freedom. Here, the most considered one is the difficulty to compute a mathematical model that approximates the robot’s behavior, e.g., kinematics and dynamics. Such a limitation does not allow the application of methods originating from the classical control theory, while the use of a simulation for the training of learning-based methods is not straightforwardly feasible. In the next two sections, a detailed description of a methodology that performs motion control without requiring a mathematical model of the robot, nor a simulation, is given.

## 3 ProMP-based methodology for non-periodic trajectories

**FIGURE 3:**
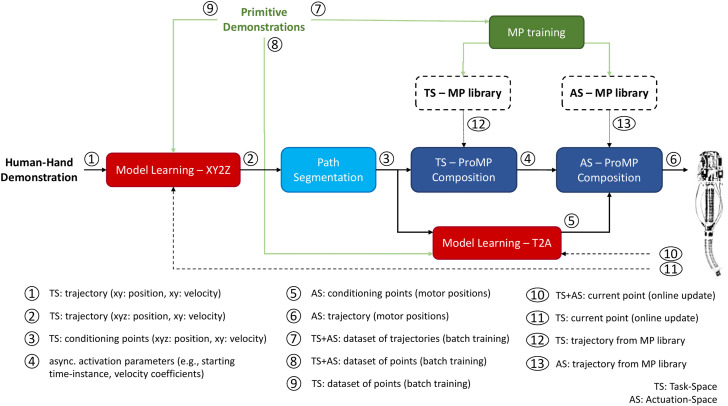
The overall architecture of the proposed controller for the reproduction of non-periodic trajectories. Blocks with solid outlines denote the various processes, while arrows indicate the flow of information between them. Specifically, green arrows and blocks correspond to the processes taking place only once (e.g., training and formulation of MP library, batch training of model learning modules), black dashed arrows constantly feed the learning-based blocks with actual measurements from the robot (e.g., EE’s position), and blocks with dashed outlines represent the MP libraries that are trained only once and their content remains unchanged. A detailed description of the functionality of each block is presented in Section 3.

The main idea behind the proposed methodology is that a requested trajectory could be described as the composition of primitives obtained by a learned MP library, and formed properly in the task-space by exploiting the ProMP’s properties. Subsequently, the composition parameters - computed for the task-space during the former step - could be transferred unchanged to the actuation-space and applied to the corresponding primitives. A block diagram is illustrated in Figure 3, briefly describing the control flow.

For the rest of this section, the task-space is comprised by the position of robot’s tip, while the actuation-space consists of the angular position of the (six) motors; hence, the actuation and the motor spaces coincide in the frames of this work.

### 3.1 Generation of primitive demonstrations and MP training

The extraction of demonstrations and consequently the formation of the ProMP library derived after training, are crucial for the proposed methodology’s performance. In the scenario of washing the human back and its involving sub-processes such as the water pouring task, the motion of the robot is limited to the quasi-plane defined by the human back’s surface. At the same time, the movement on the perpendicular direction is considered to be negligible. Accordingly, a grid of primitives built across a 2D manifold in the robot’s workspace is required, providing the capability to plan and reproduce trajectories as a result of primitives’ composition.

The process through which the demonstrations are generated is quite similar to the one described in Oikonomou et al. (2021). Concretely, a subspace *W*_*Sub*_ within the robot’s workspace is initially defined as a region of interest where the demonstrations should lie in. Subsequently, all motors are fed with actuation that result in the EE’s movement from a starting point lying on the border of *W*_*Sub*_ towards another one. Note that, at this stage the actuation corresponding to the starting and the ending point of each trajectory, is derived through experimentation in the actuation-space of the robot, and no method for position control is applied. In addition, randomness between demonstrations is enhanced by forcing the motors to pass through some median random points, lying between the two extremes, while smooth transition between consecutive points is guaranteed with linear interpolation. Therefore, a trajectory is derived as the coordinated activation and motion of all motors simultaneously. Keep in mind that, for each demonstration the sequence of motor positions (six motors, thus six sequences) and the sequence of EE’s positions in the task-space are captured along with the corresponding timestamps.

**FIGURE 4:**
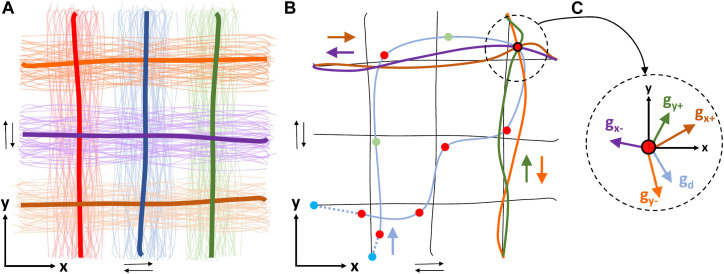
**(A)** Example of primitive demonstrations (lines in pale colors) in task-space, running in parallel with *x* and *y*-axes in both directions (towards negative and positive), covering the desired subspace of the robot’s workspace. Demonstrations of the same color are grouped according to proximity-based criteria and are trained forming a probabilistic movement primitive (mean trajectory in thick line). For simplicity, only demonstrations and primitives of a single direction (e.g., towards positive) are illustrated. **(B)** An illustrative example showing the outcome of some algorithmic components (Path Segmentation and ProMP composition in task-space) in the task-space using the grid of primitives formed in **(A)** (black lines). The cyan solid line indicates the demonstrated trajectory captured using a 3D magnetic tracker attached to the user’s hand, the cyan dashed lines connect it to the proper primitives’ edge points, while the circles (o) denote the conditioning points [Red: CPT, Green: where distance between consecutive red exceeds a threshold, Blue: artificial conditioning points lying on the *W*_*Sub*_]. Focusing on the upper right conditioning point, the four closest primitives (one for each direction) are forced to pass through it with a reference velocity. **(C)** For the conditioning point of **(B)**, the desired velocity ${\vec{g}}_{d}$ should be computed as a linear combination of the reference velocities of the corresponding primitives: ${\vec{g}}_{x+}$ , ${\vec{g}}_{x-}$ , ${\vec{g}}_{y+}$ and ${\vec{g}}_{y-}$ . The contribution of each primitive in approaching the desired velocity is computed using the simplex method.

In contrast to Oikonomou et al. (2021) where the MP library is formed by demonstrations of a single direction, here the aforementioned process is executed four times so that the resulting MPs cover all directions of the subspace and lie without loss of generality on the *xy*-plane of a local subspace frame - *S*_*D*_ = {*x* +,*x* −,*y* +,*y* − }, as shown in Figure 4A. The collected trajectories are grouped into classes under a similarity criterion, before proceeding to the MP training. In the present case, it is assumed that all demonstrations generated by a distribution around the same extreme - starting and ending - points in the actuation-space, belong to the same group. Then, the task demonstrations of the same group are trained into a probabilistic movement primitive, following the process described in Paraschos et al. (2013). Similarly, the corresponding demonstrations of the actuation-space are classified into the same groups, and are also trained into probabilistic movement primitives. Eventually, a grid of primitives is formed as shown in Figure 1, Figure 4A.

### 3.2 Human-hand demonstration

The goal of this work is to qualitatively reproduce a demonstrated trajectory using a soft-robotic arm, and evaluate it taking into consideration only the similarity on *x* and *y*-axes. The demonstrated motions are captured using a 3D magnetic tracker which is attached to the user’s hand - the hand is mainly moved on the xy-plane, while the movement on the *z*-axis is negligible. The sequence of hand velocities during the demonstrated motion are calculated in post-processing.

Before using the demonstration as input to compute the controller’s parameters, a set of pre-processing steps takes place in order to ensure that the trajectory’s execution is feasible by the robot. The first step is to scale it onto the xy-plane so that it lies within the limits of the subspace *W*_*Sub*_ defined in Section 3.1. Subsequently, for each (x,y) pair, the corresponding z coordinate that sets a feasible target in 3D-space for the robot should be estimated, using the method described in Section 3.3. The last change in the trajectory concerns its scaling with respect to the desired total duration of the execution.

At this point, it should be noted that the robot’s motion must start and finish at points lying on the border of subspace *W*_*Sub*_. This must hold due to the requirement that the proposed controller be only able to reproduce motions that are similar to the primitives. Hence, requesting zero velocity at a point located somewhere else other than the *W*_*Sub*_’s border is not feasible. As a consequence, two additional points *p*_*CS*_ and *p*_*CE*_ should be determined where the robot’s motion will start from and stop at, respectively - both should be located on the limit of *W*_*Sub*_, ensuring zero velocity. Such a derived path is depicted in Figure 4B.

### 3.3 Model learning for inverse kinematics

In cases where the complexity of the robot’s structure prevents the straightforward computation of, e.g., the inverse kinematics, alternative solutions are recommended, like the ones reviewed in Nguyen-Tuong and Peters (2011) and Sigaud et al. (2011) focusing on model learning. Nevertheless, most of these methods lack the ability to adjust on-the-fly their behavior, providing only offline training. This is a serious drawback in the context of bio-inspired systems since changes in robots’ dynamics constitute a usual phenomenon during their operation. Since the existing methods are judged inadequate to approximate the inverse kinematics, alternative solutions have been sought. Two properties that the implemented algorithm should be equipped with are: (a) the ability to exploit the total available information when this is received as data-stream, and (b) the ability to adjust online its parameters in order to be compliant with potential changes in the robot’s dynamics that may occur due to long-term use.

Focusing on data-driven methods, in Gijsberts and Metta (2013) a novel approach is presented, called Incremental Sparse Spectrum Gaussian Process Regression (I-SSGPR), lying within the general category of model learning algorithms. The authors of this work capitalised on the exhaustively studied Gaussian Process Regression aiming at designing a method that cope with unstructured and non-stationary environments where adaptability to changing conditions is required. At the same time, low computational complexity is achieved, while automated hyperparameter optimization is provided. Another interesting feature of this approach is the capability to perform both offline training using an existing dataset, as well as online updates when new data are available.

In the frames of this work, the I-SSGPR method provides an approximation of the inverse kinematics of the robot. Particularly, two separate modules based on I-SSGPR have been implemented. The first one provides a feasible target for the robot by computing the z coordinate when a {*x*, *y*}-pair is received, as explained in Section 3.2. The second module is used for the mapping of only the conditioning points (derived from the path segmentation algorithm - Section 3.4) from the task to the actuation-space. Hence, it receives the desired position (*x*, *y*, *z*) of the robot’s tip, whose z component has been computed by the former I-SSGPR module, and outputs the (six) motors’ angular positions. Both modules are initially trained offline using the dataset derived during the demonstration generation process described in Section 3.1. Later on, online updates are performed during trajectory execution by the robot, as depicted in Figure 3.

### 3.4 Path segmentation

In Oikonomou et al. (2021), the conditioning points through which the robot’s EE was requested to pass were hard coded. In contrast, here they are extracted automatically through a path segmentation process. This procedure is assigned with the task to optimally divide the path into linear segments according to a similarity criterion. The points defining the segments are then used by the controller as conditioning points. Path segmentation is the algorithmic component that follows the capturing of the motion performed by the user’s hand, and its transformation into the robot’s workspace. Before proceeding, it is noticeable that the transformed paths lie within a 2D manifold, hence 2D path segmentation algorithms are considered.

In Edelhoff et al. (2016), the authors present a variety of algorithms for segmentation of paths lying on a plane with application to animal movement patterns’ change detection, ranging from time-to topology-based methods. Focusing on the second category, the Change Point Test (CPT) method (Byrne et al., 2009) suits well in our work, since it detects significant changes in the observed movement direction (orientation). By applying this algorithm to the path extracted by the human’s hand, a set *S*_*CP*_ of conditioning points is derived.

The conditioning points obtained by the aforementioned procedure are not the only ones determined. The designed implementation considers also the case where the distance *h* between two consecutive points exceeds a predefined threshold *h*_max_. In this condition, intermediates are added to set *S*_*CP*_, the number of which is proportional to the fraction ⌊*h*/*h*_max_⌋. Eventually, the two corner points *p*_*CS*_ and *p*_*CE*_ defined in Section 3.2 are also added to set *S*_*CP*_.

Apart from its position, each point in set *S*_*CP*_ is accompanied by its velocity, as this is determined by the captured motion of the user’s hand. As for the trajectory’s corner points, an alternative velocity is hard coded in place of the zero velocity that was captured; regarding the first trajectory’s point, its velocity’s direction is set equal to that of the next trajectory’s point, while its magnitude is set equal to that of the next point of *S*_*CP*_. We proceed similarly for the last trajectory’s point. It is evident that zero velocity is also hard coded at the two corner points *p*_*CS*_ and *p*_*CE*_ of *S*_*CP*_. An illustrative example of path segmentation is depicted in Figure 4B.

### 3.5 ProMP composition in task-space and skill transfer to actuation-space

As already stated, the main idea behind this work is that the qualitative reproduction of a complex demonstrated trajectory could be accomplished with the asynchronous activation and combination of movement primitives drawn from a previously learned library.

#### 3.5.1 ProMP combination for each conditioning point

Meeting the condition of passing through the sparsely defined conditioning points is not the sole requirement; it is also crucial to achieve the appropriate velocity at a specific time. To cope with such a challenge, each conditioning point should be handled independently from the others, and hence trigger the activation of selected primitives with the required features - conditioning and duration. The transition between the primitives of consecutive points is realized with the replanning property, initially introduced in Oikonomou et al. (2021) but further extended in Oikonomou et al. (2022) (Section 3.5.2).

The procedure outlined below applies to each conditioning point *p*_*i*_ in *S*_*CP*_, with the exception of the two corner points *p*_*CS*_ and *p*_*CE*_. First, *p*_*i*_ is assigned to a primitive in each direction {*x* +,*x* −,*y* +,*y* − } based on its proximity to the closest point - this results in a classification of *p*_*i*_ into four primitives. As depicted in Figure 4B, all primitives to which *p*_*i*_ is assigned, are executed in the task-space, passing through *p*_*i*_ where the conditioning property is applied. Conditioning is also applied to the corner points of the mean trajectory for each primitive, while a reference duration *d*_*ref*_ of primitives’ execution is selected.

The purpose here is to compute how much slower or faster with respect to *d*_*ref*_ a primitive should be executed, so that the linear combination of all velocities at point *p*_*i*_ results in the desired one - note that the duration is inversely proportional to the velocity. The velocities *g*_*m*_ with *m* = {*x* +,*x* −,*y* +,*y* −,*d*} depicted in Figure 4B could be written as follows:

$$\begin{array}{cc} {\vec{g}}_{x+} & =a_{x+}\hat{x}+b_{x+}\hat{y}\\ {\vec{g}}_{x-} & =a_{x-}\hat{x}+b_{x-}\hat{y}\\ {\vec{g}}_{y+} & =a_{y+}\hat{x}+b_{y+}\hat{y}\\ {\vec{g}}_{y-} & =a_{y-}\hat{x}+b_{y-}\hat{y}\\ {\vec{g}}_{d} & =a_{d}\hat{x}+b_{d}\hat{y} \end{array}$$ (1)

where *a*_*m*_ and *b*_*m*_ are the projections’ coefficients of *g*_*m*_ on axes *x* and *y* respectively. The desired velocity *g*_*d*_ is defined as the linear combination of velocities *g*_*n*_ with *n* = {*x* +,*x* −,*y* +,*y* − } as follows:

$${\vec{g}}_{d}=l_{x+}{\vec{g}}_{x+}+l_{x-}{\vec{g}}_{x-}+l_{y+}{\vec{g}}_{y+}+l_{y-}{\vec{g}}_{y-}$$ (2)

The goal here is to find the coefficients *l*_*n*_ for which Eq. (2) holds. It should be noted that, since each velocity is derived from a primitive with a specific direction, negative coefficients *l*_*n*_ are not allowed. In this way, a system of linear equations is formulated that requires non-negative solutions, constituting a linear programming (LP) problem.

A common technique that treats such constrained systems is the simplex method described in Dantzig (2016). Initially, two new artificial variables are introduced, as the number of equations derived by Eq. 2. Proceeding to the solution, $L={\left[l_{x+},l_{x-},l_{y+},l_{y-},l_{1},l_{2}\right]}^{T}$ is requested that minimizes the linear objective function *c* ^*T*^ *L* with respect to *L*, where *c* = [0,0,0,0,1,1]^*T*^, subject to *A*_*eq*_ *L* = *b*_*eq*_ and *L* ≥ 0, where $b_{eq}={\left[a_{d},b_{d}\right]}^{T}$ and

$$A_{eq}=\left[\begin{array}{cccccc} a_{x+} & a_{x-} & a_{y+} & a_{y-} & 1 & 0\\ b_{x+} & b_{x-} & b_{y+} & b_{y-} & 0 & 1 \end{array}\right]$$ (3)

Based on simplex method, coefficients *l*_*x*+_, *l*_*x*−_, *l*_*y*+_ and *l*_*y*−_ are derived. They determine how much slower/faster the corresponding primitive should be executed with respect to its reference velocity *g*_*n*_ at point *p*_*i*_ so that the desired velocity is accomplished. As a result, each primitive’s duration is computed by $d_{n}^{\left(i\right)}=d_{\mathrm{ref}}/l_{n}$ . Given the duration $d_{n}^{\left(i\right)}$ , the last parameter that is deduced is the starting time-instance $t_{n}^{\left(i\right)}$ of each primitive in the global time-frame.

**FIGURE 5:**
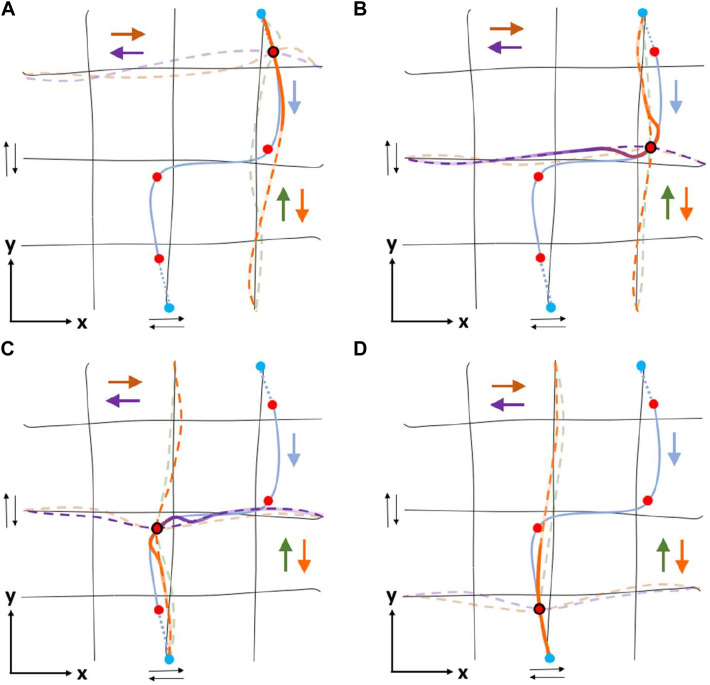
A simple example of ProMP composition at each conditioning point aiming at passing through them with proper velocity. The black lines represent the grid of mean primitives formed in Figure 4, the cyan line indicates the trajectory that the proposed methodology intends to approach and circles denote the conditioning points (Section 3.4), while colored arrows show the direction of the corresponding trajectories - black arrows imply the existence of primitives in both directions of *x* and *y*-axes. At each one of the following cases **(A)**–**(D)**, four primitives (colored dashed lines) are conditioned to pass through the conditioning point and the simplex method is applied; the output of simplex method is implied by the color intensity of each selected primitive - high color intensity means high contribution in the linear combination of primitives, and *vice versa*. On the other hand, the colored solid line indicates the trajectory derived after ProMP composition for the examined conditioning point, while line’s color fading denotes the power at each point of trajectory, defined by transition coefficient *b*_*i*_ (Section 3.5.2). **(A)** According to simplex method, only orange primitive is sufficient to approach the velocity at the first conditioning point (upper-right), thus the orange primitive and the resulted trajectory coincide. The power of the last one gradually decreases as approaching the next conditioning point. **(B–C)** For the second and third conditioning points two primitives - orange and purple - are required. In both cases, the power of the resulted trajectory remains non-negligible only around the examined conditioning point. **(D)** Similar to **(A)**, only orange primitive is sufficient to satisfy the requirements for the last conditioning point.

Therefore, the composition of multiple ProMPs over conditioning point *i* results in the following equation:

$$v_{i}\left(t\right)=a_{i}\underset{j}{\sum }u_{i,j}\left(t-t_{j}^{\left(i\right)}\right)$$ (4)

where *j* denotes the direction {*x* +,*x* −,*y* +,*y* − } of the selected primitive, *u*_*i*,*j*_(*t*) is the value of the corresponding primitive at time-instance *t*, $t_{j}^{\left(i\right)}$ refers to the starting time of primitive *u*_*i*,*j*_ at the global time-frame, and *a*_*i*_ = 1/*n*_*i*_ where *n*_*i*_ denotes the number of active primitives that contribute to conditioning point *i*. Even though four primitives are used for approximating the position and velocity at each conditioning point, simplex method might result in zero velocity for some of them, that means no contribution. In Figure 5, an example of ProMP composition at each conditioning point of a demonstrated trajectory is depicted.

Subsequently, the skill transfer process is performed and all parameters derived for the composition of primitives in the task-space, such as $d_{n}^{\left(i\right)}$ , are transferred unchanged to the actuation space. At the same time, the MP library - where the mapping at the primitive level between the two spaces is stored (Section 3.1) - provides the corresponding primitives in the actuation-space, while the I-SSGPR module (Section 3.3) interprets all conditioning points into motor commands.

#### 3.5.2 Replanning at the ProMP level

The replanning process at the primitive level is introduced in Oikonomou et al. (2021) in order to cope with the constant changes of the conditioning points in the actuation space, occurring when the learning-based controller is updated. Hence, the resulting trajectory is always compliant with the new estimation, performing necessary changes online rather than after the end of the execution.

In a similar manner, the replanning property is also exploited in the frames of this work. Here, they handle the transition between primitives of consecutive conditioning points by gradually decreasing the power of the last primitives, while increasing the power of the next ones. It should be clarified that replanning differs from blending, in the sense that the primitives on which it is applied are not necessarily executed in parallel under synchronous activation, as it is assumed in Paraschos et al. (2013). Additionally, in this application the intuition behind replanning is that it ensures smooth transition between sequential primitives formed by consecutive transition points, rather than blending them.

**FIGURE 6:**
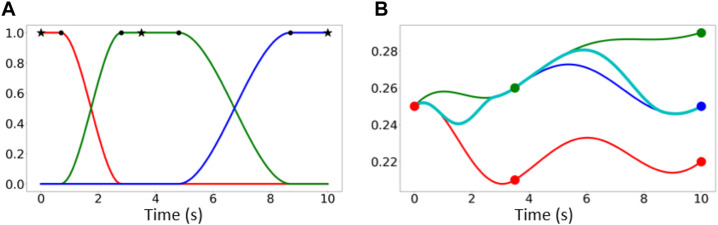
**(A)** *Y*-axis denotes the weight $\left[0,1\right]$ of the corresponding primitive, while stars (*) indicate way-points, and circles (o) the time-interval where transition function *b*_*i*_(*t*) remains fixed. **(B)** The activation of Red-Green-Blue trajectories is indicated by the corresponding *b*_*i*_(*t*) in **(A)**, while the Cyan is the resulting trajectory generated through replanning. Here, the *y*-axis might denote either the trajectory in one axis of the task-space, or the angular position of a motor in the actuation-space.

**FIGURE 7:**
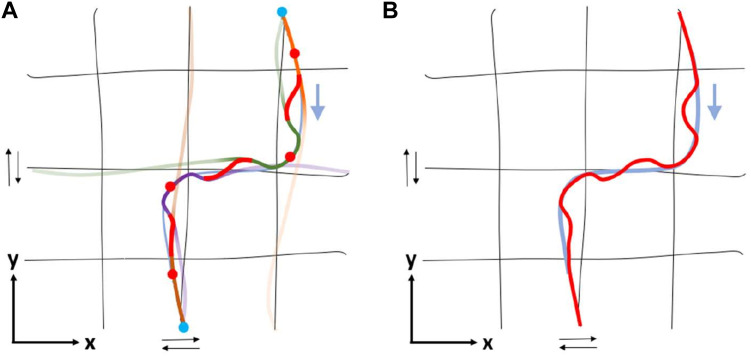
Continuing from Figure 5: Transition between trajectories of consecutive conditioning points through replanning in the *xy*-plane (task-space). Note that, in this example the deviations from the real trajectory are purposefully exaggerated to illustrate the transition between conditioning points. The deviation shown could be reduced either by increasing the number of conditioning points, or by conditioning on several consecutive points. Nevertheless, as can be seen in Figure 8 in practice even with a small number of conditioning points we obtain small deviations. **(A)** The trajectories derived for each conditioning point following the process described in Section 3.5.1 are here connected using functions that ensure smooth transitions (red lines). Each trajectory is illustrated by a solid line, whose color fading denotes the value of the corresponding transition function; faint color implies negligible contribution to the replanned motion. **(B)** Total resulted (red) vs demonstrated (light blue) trajectory.

Proceeding from Eq. 4, the resulting trajectory *v*(*t*) is determined as follows:

$$v\left(t\right)=\overset{N}{\underset{i=1}{\sum }}b_{i}\left(t\right)v_{i}\left(t\right)=\overset{N}{\underset{i=1}{\sum }}b_{i}\left(t\right)\left[a_{i}\underset{j}{\sum }u_{i,j}\left(t-t_{j}^{\left(i\right)}\right)\right]$$ (5)

where *b*_*i*_(*t*) denotes the transition function for conditioning point *i* and *N* refers to the total number of conditioning points. An example of a transition function *b*(*t*) along with the corresponding replanned trajectory in a one dimensional motion is illustrated in Figure 6. Figure 7 depicts the trajectory derived from Figure 5 after replanning.

Eventually, given that all primitives are formed, the execution takes place. They are activated independently and asynchronously as determined by their starting time-instance $t_{n}^{\left(i\right)}$ , while the replanning property handles the transition between primitives of consecutive conditioning points.

### 3.6 Performance demonstration

The capabilities of each algorithmic component as well as the performance of the methodology as an entity for the reproduction of non-periodic trajectories were analytically evaluated in Oikonomou et al. (2022) through an experimental process conducted using the soft-robotic arm described in Section 2. Here, a brief presentation of the research findings is provided to illustrate the properties of the method, before proceeding to the introduction of the extended version of the architecture to periodic movements.

**FIGURE 8:**
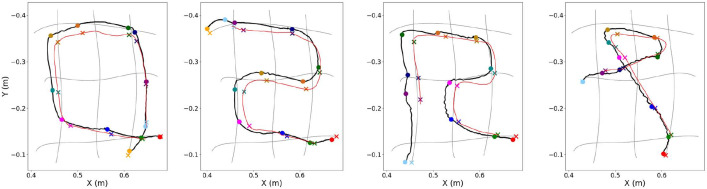
Experimental results illustrating the execution (solid black line) of four demonstrated trajectories (pale red line) by the soft-robotic arm. The desired conditioning points are depicted with colored x symbols, while the executed points at the same timestep are depicted with the respective colored dots. The red symbols (x and dot) indicate the first conditioning point of the trajectory. Adapted from Oikonomou et al. (2022), with permission from IEEE.

As shown in Figure 8, the proximity level between the desired and the performed trajectories implies that the proposed methodology has the capability to qualitatively reproduce demonstrated hand’s motions, since it manages to handle the movement not only close to the conditioning points but also in the intermediate space. The spatial similarity is due to the proper composition of primitives for approximating not only the conditioning points but also the velocity that was recorded at each of them.

Importantly, this method enables generalization to the production of circular and diagonal trajectories (Figure 8) that are different from the initially learned library of horizontal and vertical MPs. Finally, it is interesting to note that this methodology provides the capability to execute periodic trajectories while handling them as if they were non-periodic. Nevertheless, its inefficiency over the proposed extension is shown in Section 5.3, where the experimental comparison of the two methods are presented.

## 4 Extension for periodic trajectories

**FIGURE 9:**
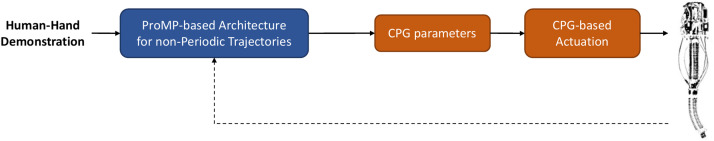
The overall proposed architecture for the reproduction of periodic trajectories as extension of Figure 3. The first block (blue) consists of all processes taking place in case of non-periodic trajectories (Section 3), while the next two processes are required for transforming the ProMP-based actuation into CPG-based. Black dashed arrow constantly feeds the learning-based blocks with actual measurements from the robot. A detailed description of the functionality of each one of the new blocks is presented in Section 4.

The methodology described in Section 4 provides the soft robot with the capability to perform arbitrary non-periodic trajectories in a model-free zero-shot manner. The main novelty here consists of using the same methodology as a pre-computational process in a modified architecture in order to facilitate the execution of rhythmic patterns. Such an extension is activated by adding two additional processes at the end of the previous methodology (Figure 3), as depicted in Figure 9.

### 4.1 Justification on the extension’s necessity

Normally, a periodic phenomenon has neither discrete start nor end points that determine its duration and thus its existence. On the contrary, there are a quasi-start and a quasi-end points that coincide, while all the others appear repeatedly after a specific duration, which is eventually called period. The method described in the previous section requires that the desired trajectory is defined within some specific temporal limitations, e.g., a predetermined duration, so that its computation is feasible. However, in cases where the targeting motion is periodic and thus its start and end points are not explicitly defined, the reproduction task should be handled differently. The goal of the proposed extension of the control architecture is the simplification of the trajectory execution under the presence of a repeated pattern.

### 4.2 Methodology extension

The second important novelty of this work consists in compressing a learned periodic movement into the parameters of a CPG. CPGs are neural circuits found in animals’ nervous system that are capable of producing rhythmic coordinated patterns of high-dimensional output signals out of simple low-dimensional input signals (Ijspeert, 2008). Their interesting properties led to the design of neurobiological mathematical models that approximate their functionality. In Oikonomou et al. (2020) the authors exploited CPGs’ capabilities to modulate the features of the produced rhythmic output (e.g., frequency) by using simple control signals, aiming at the reproduction of periodic movements of desired features. Likewise, the present work focuses on utilizing the property of the CPG to generate complex signals out of simple commands, as well as the ability of a soft robot to produce complex motions due to its dexterity. As already stated, the main strength of the proposed approach lies in its ability to avoid the time-consuming training process required in Oikonomou et al. (2020), while providing a zero-shot learning approach. The actuation which is finally received by the motors of the robot is similar to the one computed in Oikonomou et al. (2020).

Before proceeding to the presentation of the two additional processes, note that in this work the desired trajectory is a parameterizable ellipse lying on the xy-plane. Such a two dimensional periodic motion can be decomposed into two single-frequency oscillations - one on each axis, determined by an offset, an amplitude and a phase bias. While the elliptical shape of the target trajectory has been chosen for simplicity, the proposed methodology might be similarly applied to any trajectory that constitutes a linear composition of multiple oscillations in each dimension.

In addition, here, a less sophisticated implementation of path segmentation takes place which is permitted due to the periodicity of the desired trajectory. The latter is divided into *n*-segments within a period of motion, which implies that a conditioning point is hard-coded every 2*π*/*n* radians. This modification has minor effects on the outcome compared to the CPT approach. Nevertheless, various numbers of conditioning points within a period are tested and evaluated as shown in Section 5.

The contribution of the new blocks adding the capability of reproducing periodic trajectories (Figure 9) is described below.

#### 4.2.1 Computation of CPG-parameters

This module follows the ProMP-based part of the methodology, and its goal is to detect the intrinsic periodicity of the actuation signals along with their features, and subsequently to decompose it into discrete single-frequency oscillations. Here, the first step is the isolation of the oscillatory part from the resulting signal which contains also the actuation performing the transition from the border of the primitive’s grid to the edge of the requested trajectory (see Section 3.2 and Figure 4B). The periodic signal can be easily obtained since the timestamps of the conditioning points are already known. Subsequently, the simplest way to compute a periodic signal’s components is by applying Fast Fourier Transformation (FFT) (Cooley and Tukey, 1965). Through this process the original actuation is filtered since only the dominant frequency is kept, and eventually the computed features - offset, amplitude and phase bias - for each motor are promoted to the next block, where a CPG-based reconstruction of the actuation takes place.

#### 4.2.2 CPG-based actuation

In this approach, the motors of the soft-robotic arm can be seen as a system of six coupled oscillators (one for each motor) that produce appropriate rhythmic signals, resulting in the generation of periodic movements by the robot’s tip. In contrast to Oikonomou et al. (2020) where the offset of the signal was not included in the control parameters, here each oscillator is parameterizable in terms of frequency, amplitude, offset, as well as phase difference with respect to the signal of a motor that is predefined as reference. The redefinition of control parameters allows the change of the center around which the motion takes place. The implemented CPG model is mathematically formulated by the following system of equations (Crespi et al., 2008):

$$\begin{array}{cc} \dot{\phi_{i}} & =\omega_{i}+\underset{j}{\sum }\left(w_{ij}r_{j}\sin\left(\phi_{j}-\phi_{i}-\varphi_{ij}\right)\right)\\ \ddot{r_{i}} & =\alpha_{r}\left(\frac{\alpha_{r}}{4}\left(R_{i}-r_{i}\right)-\dot{r_{i}}\right)\\ \ddot{x_{i}} & =\alpha_{x}\left(\frac{\alpha_{x}}{4}\left(X_{i}-x_{i}\right)-\dot{x_{i}}\right)\\ \theta_{i} & =x_{i}+r_{i}\cos\phi_{i} \end{array}$$ (6)

where the state variables *ϕ*_*i*_, *r*_*i*_ and *x*_*i*_ represent the phase, the amplitude and the offset of the *ith* oscillator, respectively; these are computed iteratively using a numerical method that allows us to approximate solutions to differential equations. Besides, the parameters *ω*_*i*_, *R*_*i*_, *X*_*i*_ and *φ*_*ij*_ provide control over the frequency, amplitude, offset as well as the phase biases between oscillators *i* and *j*, respectively; they are determined by the decomposition of the actuation signals performed in the previous step through the FFT algorithm. The phase biases *φ*_*ij*_ along with the weights *w*_*ij*_ define the coupling between the oscillators *i* and *j*. Eventually, *α*_*r*_ and *α*_*x*_ are positive constants, while *theta*_*i*_ is the rhythmic output signal extracted from oscillator *i*.

## 5 Experimental evaluation

Although multiple algorithms are utilized in the overall control architecture, the evaluation is limited to the contribution of the proposed extension. The evaluation taking place in this section focuses mostly on highlighting the advantages and the efficiency of our implementation over the previously developed methods introduced in Oikonomou et al. (2020); Oikonomou et al. (2022). Towards this direction, a set of experiments is conducted on the real soft arm, and a qualitative comparison between them is attempted, in order to further exhibit its usefulness in the present application.

From now on, to provide a clear distinction through the rest of the manuscript between the various methods, the approach introduced in Oikonomou et al. (2020) is referred to as CPG-based, the one in Oikonomou et al. (2022) as ProMP-based, while the extension proposed in this paper is denoted by the abbreviation MP-D2P (Movement Primitive - from Discrete To Periodic).

### 5.1 Learning the ProMP library and the I-SSGPR modules

Initially, directed demonstrations are generated by the robot while a magnetic tracker attached on the robot’s tip was capturing not only the generated paths in the task-space, but also the sequence of actions. Then, the demonstrations recorded in both task and actuation spaces are grouped into classes and trained to form the MP library following the process described in Section 3.1. Additionally, the data collected during the aforementioned process - the robot tip’s positions along with the corresponding actuations - are exploited to form the dataset *D*_*pod*_, which is used to offline train the two I-SSGPR modules as described in Section 3.3.

In the frames of the present work, ten trigonometric basis functions are used to construct each I-SSGPR module. Right after the end of the offline training session, their performance is assessed based on how well they approximate the training dataset *D*_*pod*_. Starting from the module that computes the z-coordinate for a {*x*, *y*}-pair ensuring a feasible target inside the robot’s workspace, the computed error is [3.5 ± 1.4] (mm). Moreover, the module that approximates the inverse kinematics, by receiving the {*x*, *y*}-pair along with the previously computed coordinate on the *z*-axis as input and outputs the corresponding actuation, results in [535 ± 316] (ticks) error measured for each motor. Taking into account that 32,768 ticks are equivalent to one full periodic rotation of the motor shaft, then the aforementioned error is translated to [2.5 ± 1.5] (mm) error in cable length. This corresponds to 5% of the robot’s operational workspace, because the range of change for each cable is more than 50 mm. This error is low considering the stochasticity induced by the mechanical structure of the soft robot.

From now on, the learned models (ProMP library and I-SSGPR modules) are used as knowledge base by the planner in order to perform some desired tasks. On the one hand, the content of the ProMP library for both the actuation and the task-space is fixed. Meanwhile, the I-SSGPR modules are constantly updated as new data are obtained in the course of the robot’s execution.

The experiments conducted on the real robot aim mainly at assessing both qualitatively and quantitatively the performance of the proposed extension, and subsequently at demonstrating its enhanced capabilities.

### 5.2 Varying number of conditioning points

**FIGURE 10:**
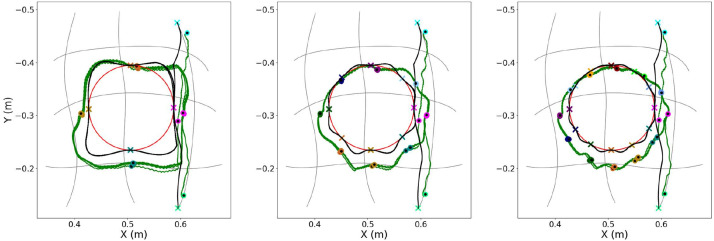
Execution of the same trajectory for three different numbers of conditioning points per period using the ProMP-based approach; from left to right: {4, 8, 12} conditioning points. Each figure depicts the targeted trajectory (red line), the planned motion computed as a blending of primitives through replanning (black line), as well as the execution of the planned actuation by the soft-robotic arm for five periods (green line). The desired conditioning points are depicted with colored x symbols, while the executed points at the same timestep are depicted with the respective colored dots. The cyan symbols (x and dot) at the border of the primitives’ grid indicate the first conditioning point of the trajectory.

**FIGURE 11:**
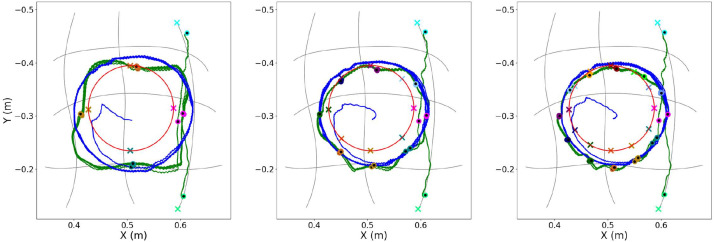
Continuing from Fig. 10, here is the execution as this is computed and performed by MP-D2P (blue line). Similarly, the number of conditioning points per period are, from left to right: {4, 8, 12} conditioning points. The desired trajectory (red line) as well as the executed one by the ProMP-based approach (green line) are also included for comparison purposes.

Initially, an evaluation is attempted on how the number of conditioning points affects the planning of the primitives’ blending and the computation of the requested actuation. The results obtained from the first experimental session are depicted in Figure 10 and Figure 11. There, all trajectories are illustrated for three different numbers of conditioning points per period: {4, 8, 12}.

In Figure 10, each plot contains the desired path along with the conditioning points, the planned one computed as a blending of primitives as well as the executed one using the ProMP-based method. It can be easily noticed that the more conditioning points are defined on the desired trajectory, the higher the segmentation resolution, and the better the accomplished approximation. Another outcome that is extracted from the 2D plots in the same figure is that the planned trajectory (black line) is relatively close to the executed one (green line) for all three cases. This implies that the mapping at a primitive level provided by the MP library is sufficient for allowing the planning in the task and the subsequent transfer to the actuation-space. In addition, the mean error at a conditioning-point level, in terms of both position and velocity, is also small after five executions, as seen in Table 1. The table indicates the capability of I-SSGPR modules to approximate adequately the inverse kinematics. At the same time, the corresponding standard deviation included in the same table is non-zero due to the stochasticity induced by the soft properties of the robot. Nevertheless, standard deviation is still relatively small, which reveals the good repeatability of the gestures executed by the robot.

**TABLE 1:** Mean error measurements for five (5) consecutive executions of the periodic trajectories by the soft-robotic arm, depicted in Figure 10. The position error *e*_*p*_ (cm), the velocity direction error $e_{{\hat{v}}_{xy}}$ (deg) and the ratio of the actual velocity to the desired one $e_{{\left|\vec{v}\right|}_{xy}}$ (dim/less) are all measured in each conditioning point **CP##** at the corresponding time-step. The last column computes the average of standard deviations for all conditioning points.

#CP	Error type	CP_*S*_	CP01	CP02	CP03	CP04	CP05	CP06	CP07	CP08	CP09	CP10	CP11	CP12	CP_*E*_	std
**4**	***e*_*p*_ (*cm*)**	2.8	2.3	2.6	1.7	1.3	-	-	-	-	-	-	-	-	2.7	<± 0.1
	$e_{{\hat{v}}_{xy}}\left(\deg\right)$	-	6.5	1.7	24.8	10.8	-	-	-	-	-	-	-	-	-	±1.1
	$e_{{\left|\vec{v}\right|}_{xy}}$	-	1.24	1.15	0.99	1.19	-	-	-	-	-	-	-	-	-	±0.03
**8**	***e*_*p*_ (*cm*)**	2.3	2.9	2.6	2.9	2.5	1.9	0.6	1.5	2.9	-	-	-	-	3.0	<± 0.1
	$e_{{\hat{v}}_{xy}}\left(\deg\right)$	-	11.0	17.7	2.3	5.3	22.4	2.5	13.2	16.4	-	-	-	-	-	±1.2
	$e_{{\left|\vec{v}\right|}_{xy}}$	-	1.29	1.29	1.35	1.50	1.04	1.22	1.18	1.15	-	-	-	-	-	±0.02
**12**	***e*_*p*_ (*cm*)**	2.3	2.9	2.4	2.7	3.2	3.0	2.3	2.2	1.2	0.8	1.1	2.1	2.6	3.0	<± 0.1
	$e_{{\hat{v}}_{xy}}\left(\deg\right)$	-	3.2	8.8	3.7	4.5	7.6	1.6	5.5	6.3	4.9	5.5	1.4	3.1	-	±0.9
	$e_{{\left|\vec{v}\right|}_{xy}}$	-	1.16	1.21	1.26	1.15	1.41	1.06	1.18	1.23	1.36	1.22	1.16	1.18	-	±0.03

Figure 11 enables to visually compare the results of the ProMP-based method and MP-D2P proposed in this paper. In the figure, each plot illustrates the desired path along with the conditioning points, the executed one using the ProMP-based method and the one resulted from MP-D2P. Before proceeding to a more quantitative comparison, the assessment of the new method takes place in terms of how well it approximates the desired periodic trajectory. However, in this case the evaluation of the performance based on the error measured at the conditioning points is not appropriate; actually, focusing on the accuracy at some sparse points might be sometimes misleading for extracting a safe outcome regarding the efficiency of a method. For example, in the first scenario in Figure 10 where 4 conditioning points are used, even though the computed error at each one is small, the executed trajectory differs largely from the desired one. Therefore, the evaluation here is based on the parameters - offset, amplitude and phase bias - of the dominant frequency’s component for the two motions - the desired and the executed one. In Table 2 the resulting parameters are presented after decomposing each trajectory into two oscillations, one on the *x*-axis and one on the *y*-axis. The relatively small errors shown there along with the visual similarity depicted in Figure 11 demonstrate the capability of the proposed method to qualitatively reproduce the specific periodic motion of this example.

**TABLE 2:** Assuming that each periodic trajectory lying on the *xy*-plane consists of two oscillations - one in each axis - only five parameters are required for fully defining it; setting the *x*-axis as reference, PhaseBias_**XX**_ is omitted. This table presents the features that characterize each desired and the corresponding executed motion. ID 1 is assigned to the trajectories illustrated in Figure 11, while IDs 2 and 3 correspond to the first two trajectories depicted in Figure 12. The second column denotes whether the features represent the desired trajectory, or the number of conditioning points defined for the executed by the proposed method.

traj. ID	type	Offset_X_ (m)	Amplitude_X_ (m)	Offset_Y_ (m)	Amplitude_Y_ (m)	PhaseBias_YX_ (rad)
**1**	**desired**	0.508	0.080	−0.315	0.080	1.571
	**4 CPs**	0.511	0.111	−0.305	0.107	1.607
	**8 CPs**	0.510	0.100	−0.306	0.097	1.608
	**12 CPs**	0.511	0.098	−0.304	0.094	1.596
**2**	**desired**	0.508	0.100	−0.365	0.054	1.571
	**12 CPs**	0.508	0.122	−0.357	0.066	1.436
**3**	**desired**	0.508	0.088	−0.326	0.090	2.151
	**12 CPs**	0.511	0.108	−0.313	0.104	2.124

The two following sections, present a more focused and detailed comparison of the new approach with respect to the previously developed methods.

### 5.3 Comparison: ProMP-based approach vs MP-D2P

Although the ProMP-based methodology (Oikonomou et al., 2022) is able to qualitatively reproduce any trajectory, the proposed extension is more beneficial regarding the approximation of periodic trajectories, as indicated from the experiments. Some of the advantages that set it more preferable over the ProMP-based approach are listed below.

**FIGURE 12:**
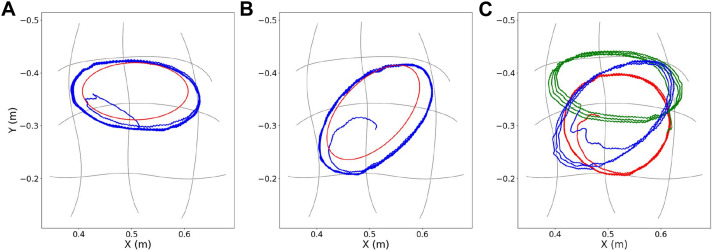
MP-D2P: **(A)**–**(B)** Execution of two periodic trajectories with elliptical shape (in blue), and illustration of the corresponding desired ones (in red). **(C)** Online transition between three periodic trajectories during execution.

**FIGURE 13:**
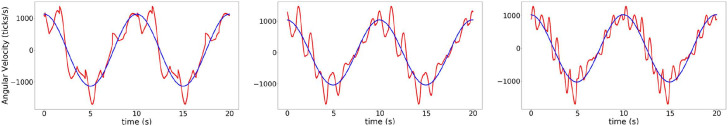
First derivative of computed actuation for the same periodic trajectory using the ProMP-based approach (in red) and the MP-D2P method (in blue), for varying number of conditioning points, from left to right: {4, 8, 12}.

(a) The ProMP-based method is highly dependent on the number of conditioning points defined prior to execution. Specifically, it is noticed that when using four points (left plot in Figure 10) the resulting trajectory largely differs from the desired one, even though the error at these points is relatively small. This is not the case for the new approach where the zero and the most dominant frequency of the actuation are only kept through filtering, deriving a closer reproduction of the desired periodic motion (Figure 11). However, even in this case more conditioning points result in better approximation.
(b) A restrictive property of the ProMP-based method is that the motion must always start from and finish to somewhere at the border of the grid of primitives where zero velocity can be accomplished. In contrast, the translation of the actuation into CPG format allows the start and end of the trajectory to take place wherever within the operational robot’s workspace.
(c) Another flaw of the ProMP-based approach is that some features, such as the number of periods requested to be executed, have to be predetermined by the user during the definition of the desired periodic motion. In contrast, the translation and the execution of the actuation by the CPG module allows the performance to be continuous until it is (manually) modified or stopped. Similarly, the use of the CPG module also ensures smooth transition to periodic trajectories of different features during the course of execution. Figure 12C illustrates in different colors the transition from one periodic trajectory to another when a proper signal is received by the motors’ controller. Both requests for online adaptation or change of the executed trajectory described here could be achieved by the ProMP-based module through the replanning process. However it constitutes a complicated procedure since it requires the definition of the transition points.
(d) Last but not least, the velocities computed by the ProMP-based approach and planned to be executed by the robot’s motors, require abrupt changes in their magnitude as shown in Figure 13 and hence big accelerations. It is also noticed that the accelerations are increased proportionally to the density and thus the number of conditioning points. On the contrary, here the actuation is previously filtered and eventually reconstructed using only a finite number of frequency components, no matter how many conditioning points are used for the approximation. Hence, the corresponding velocities have been smoothed before they are applied to the motors.

### 5.4 Comparison: CPG-based approach vs MP-D2P

Generally, the rhythmic patterns constitute complex non-linear motions. However, the controller based on CPGs facilitates its generation due to the low dimensional and easily parameterizable input signals, as explained in Ijspeert (2008). Moreover, their combination with discrete MPs and concretely the exploitation of a ProMP framework such as the one developed in Oikonomou et al. (2022), solves many limitations presented in Oikonomou et al. (2020), the most important of which are summarized below.

(a) The use of a simulation for speeding up the training process is almost impossible, due to the absence of a reliable mathematical model that adequately approximates the specific robot’s behavior. As a result, performing the whole learning procedure directly on the soft arm is the only way forward. However, this process is time-consuming, and requires exhaustive exploration of many iterations through trial-and-error for the learning-based model to converge, especially in cases of learning with no prior knowledge - from scratch. The estimation of the actuation provided by the ProMP approach, before the conversion into CPG format, accelerates significantly the computation.
(b) One of the main drawbacks of the previous implementation is that the offset of the actuation is not included in the model’s state-action space - contrary to the amplitude and the phase bias. As a consequence, the learned trajectories are limited around a fixed center. As shown in the experiments and illustrated in Figure 12, the extended methodology provides the capability of executing motions around all centers lying within the robot’s workspace.

## 6 Discussion

The goal of this work was the design of a control architecture as an extension of a previously developed method (Oikonomou et al., 2022), that is capable of qualitatively reproducing periodic trajectories of desired features applied on a modular robotic arm with soft properties. MP-D2P is based on the idea that a periodic motion in the task-space could be more efficiently controlled by rhythmic patterns employed in the actuation, such as those generated by a CPG (Ijspeert, 2008). The derived results proved that the conversion of the learned actuation with ProMPs into a CPG format provides a good approximation of the desired motion, considering the constraints in terms of accuracy induced by the robot’s mechanical structure. Its comparison with the previously developed methods shows that the MP-D2P exploits the advantages of each one of them; briefly, it requires less training than the CPG-based approach and directly provides a qualitative estimation of the actuation by exploiting a MP library used as knowledge base. In addition, it eventually computes a low-dimensional control parameterization for generating the desired rhythmic pattern.

In this work, the training process - and thus the execution and the evaluation - is focused on a subspace of the workspace in order to facilitate the analysis of the methodology. The chosen subspace is quasi-planar, hence the definition of the grid of primitives and their directions (see Section 3.1) is more straightforward in terms of implementation, while it facilitates visualization and analysis. Either way, the chosen subspace already covers most of the reachable workspace of the soft-robotic arm. To further extend it we would need to go 3D. While this is for future work (see last paragraph of this section), we have good reasons to think that this can be extended in a straightforward way to cover 3D trajectories. This requires the training of new primitives, e.g., in parallel to the *z*-axis. A potential issue in case the workspace is extended on a plane that is perpendicular to the ground, concerns the capability of the soft-robotic arm to bear large torques that might be produced by the gravitational force. However, in principle the controller should be able to manage to withstand the forces, since the trained primitives would result from trajectories that would have been generated under gravity.

The term “zero-shot” does not imply the absence of knowledge, but the fact that any unseen trajectory can be generated using a fixed library of known simple trajectories. In such a case, a relatively good approximation is achieved right from the first execution, without requiring more than one trials until the controller’s parameters converge. In this work, the library of primitive trajectories consists of quasi-straight lines, while the executed ones include turns. The research findings presented in Sections 3.6 and 5 exhibit the zero-shot learning property of the controller. In similar approaches like in Ijspeert et al. (2002), the execution of the desired trajectory using the proposed controller is preceded by the demonstration of the motion (to the robot) by a human through kinesthetic teaching. There, a sequence of actions is captured by the encoder integrated in each joint of the robot. However, such an approach is not feasible in the present application due to the soft properties of the robot’s mechanical structure. At the same time, kinesthetic teaching in most cases requires the knowledge of a model that computes the robot’s dynamics, in order to compensate gravity’s effect. Nevertheless, the proposed controller extracts the requested actuation in a model-free manner avoiding the design of complex models that may not approximate well the soft robot’s behaviour.

Moreover, the introduced control strategy may be applied to any robot without requiring the knowledge of a model that approximates the robot’s behaviour, since it constitutes a model-free method. This adds to our previous work by further showing the benefit in terms of adaptivity, flexibility, and low computational cost (compared to model-based methods) of bio-inspired model-free reinforcement learning robot architectures (Khamassi et al., 2006; 2011; Renaudo et al., 2014; Khamassi et al., 2018). Compared to this previous work, which mostly used discrete actions, or combined discrete actions with continuous movement parameters in a parameterized action space (Khamassi et al., 2017; Khamassi et al., 2018), the method proposed here enables to learn continuous, complex, and even periodic, trajectories of the robot’s arm, which further extends possible applications.

The proposed solution may offer new possibilities for the integration of soft robots into systems that serve for medical purposes, such as the water pouring task considered in this work which is part of the more general showering application. For example, in a relevant context, a batch of periodic movements selected specifically for each patient, could be demonstrated and recorded by an expert (e.g., nursery staff) and stored in a “personal” library of the individual. Subsequently, according to the washing scenario the appropriate motion would be autonomously reproduced by the robot in zero-shot manner. Such an advancement would enable automation while ensuring safety, and in the specific application might be beneficial for the clinical personnel whose workload could be dramatically reduced, as well as for the patients. In the latter case, such an autonomous system would provide comfort and a sense of independence to the users who may feel exposed in terms of privacy when other humans (e.g., therapists) intervene on their behalf.

In future work, we plan to improve the execution of the periodic motion in terms of accuracy. This will be achieve by refining the learning-based model of robot’s inverse kinematics, and by providing online correction to errors through the exploration of better actions and convergence during execution. Moreover, the extension to a 3D grid of primitives is part of the future work. Eventually, further investigations that could enhance the value of the proposed methods are those that concern the reproduction of trajectories consisting of combined translational and periodic parts. These components could be extracted automatically by decomposition of the human hand’s motion, so that the two main methodologies - ProMP-based and MP-D2P - perform simultaneously.

## Data Availability

The raw data supporting the conclusion of this article will be made available by the authors, without undue reservation.
